# The Influence of Physical Activity during Pregnancy on Miscarriage—Systematic Review and Meta-Analysis

**DOI:** 10.3390/jcm12165393

**Published:** 2023-08-19

**Authors:** Rubén Barakat, Dingfeng Zhang, Cristina Silva-José, Miguel Sánchez-Polán, Evelia Franco, Michelle F. Mottola

**Affiliations:** 1 AFIPE Research Group, Faculty of Physical Activity and Sport Sciences-INEF, Universidad Politécnica de Madrid, 28040 Madrid, Spain; barakatruben@gmail.com (R.B.); zhangdingfeng123@gmail.com (D.Z.); cristina.silva.jose@upm.es (C.S.-J.); miguelsanpol@gmail.com (M.S.-P.); 2 Department of Education, Research and Evaluation Methods, Faculty of Social and Human Sciences, Universidad Pontificia de Comillas, 28049 Madrid, Spain; 3 R. Samuel McLaughlin Foundation-Exercise and Pregnancy Lab, 2245, 3-M Centre, School of Kinesiology, Faculty of Health Sciences, Department of Anatomy & Cell Biology, Schulich School of Medicine & Dentistry, Children’s Health Research Institute, The University of Wester Ontario, London, ON N6A 3K7, Canada; mmottola@uwo.ca

**Keywords:** pregnancy, physical activity, miscarriage, exercise

## Abstract

Miscarriage is an inability to complete the normal process of pregnancy and childbirth and represents a major concern for pregnant women that can be an emotionally devastating event. While it has been suggested that engaging in strenuous physical activity might be associated with an elevated risk of miscarriage, there is a recent systematic review that suggested that prenatal exercise is not associated with fetal mortality. The aim of this systematic review and meta-analysis (SR + MA) was to assess the effects of physical activity during pregnancy on the likelihood of experiencing a miscarriage (Registration No.: CRD42022370629). Thirteen randomized clinical trials (3728 pregnant women) were included. Meta-analyses were conducted with the dependent variable being the miscarriage ratio in each study. The total risk ratio (RR) sum was calculated using a random effects model. The I^2^ statistic was utilized to quantify the heterogeneity observed in the results. No significant association between exercise during pregnancy and the occurrence of miscarriage was found (RR = 0.83 95% CI = 0.83 (0.49–1.41); z = 0.69, *p* = 0.49; I^2^ = 0.00%, Heterogeneity *p* = 0.91). Results of the present SR + MA showed no increase in miscarriage risk in those who engaged in low- to moderate-intensity exercise compared to those who did not.

## 1. Introduction

Miscarriage is an inability to complete the normal process of pregnancy and childbirth and represents a major concern for pregnant women that can be an emotionally devastating event [1]. The effects of engaging in physical activity on miscarriage risks, especially in the first trimester, have been a historical concern for both pregnant individuals and their physicians. While it has been suggested that engaging in strenuous physical activity might be associated with an elevated risk of miscarriage [2], there is a recent systematic review that suggested that prenatal exercise is not associated with fetal mortality [3]. Previous studies have also evidenced that the association between physical activity and miscarriage risks might be null [4], or, that physical activity might be a protective factor against miscarriages [5,6].

One of the barriers to physical activity is the fear of miscarriage, which may delay pregnant individuals from starting or continuing an exercise program throughout pregnancy, especially in the first trimester [7]. Since only 15% of the population is meeting current international guidelines of 150 min of physical activity per week [8], it is important that scientific evidence be reviewed to assist in alleviating these fears in order to encourage physical activity throughout pregnancy. The benefits of physical activity are numerous to both the pregnant individual and the developing fetus [9]. Nevertheless, there remains a paucity of scientific knowledge regarding the association between gestational physical activity and the risk of miscarriage. Considering that miscarriage risk is a matter of concern during pregnancy, there is a need to gain an understanding of the effects of physical activity on miscarriage risk. Consequently, a thorough review of experimental studies investigating the impact of physical activity during pregnancy on the occurrence of miscarriage is greatly warranted.

The main objective of this systematic review and meta-analysis is to assess the effects of physical activity during pregnancy on the likelihood of experiencing a miscarriage.

## 2. Materials and Methods

The current study was conducted in accordance with the Preferred Reporting Items for Systematic Reviews and Meta-Analyses (PRISMA) guidelines and was registered with the International Prospective Register of Systematic Reviews (PROSPERO, Registration No. CRD42022370629). The population, intervention, comparison, outcomes, and study design (PICOS) framework was employed to analyze the search sources [10].

### 2.1. Population

The population included pregnant individuals without any relative obstetric contraindications (e.g., gestational hypertension, malnutrition, or moderate cardiovascular disease) or absolute contraindications (e.g., premature labor, preeclampsia, or incompetent cervix) who participated in a prenatal physical activity program.

### 2.2. Intervention

Analyzed characteristics of the intervention were as follows: (a) weekly frequency of physical activity sessions; (b) intensity, where all studies included utilized a moderate load intensity, defined as 55–65% of the maximum maternal heart rate or the perceived effort on the Borg Scale (range 12–14); (c) duration of the physical activity program; (d) type of physical activity, including yoga, Pilates, aerobic exercises, strength training, or pelvic floor training; (e) supervision of the physical activity program; (f) duration of the individual sessions, as presented in Table 1.

### 2.3. Comparison

Women who participated in an exercise or physical activity program during pregnancy were compared with those who did not. Intervention characteristics were collected and compared, as presented in Table 1.

### 2.4. Outcomes

The occurrence of miscarriage served as the primary (target) outcome of interest.

### 2.5. Study Design and Selection Process

The literature search was conducted between September and November 2022 at Universidad Politécnica de Madrid (INEF), using the following databases: EBSCO, including Academic Search Premier, Education Resources Information Center, MEDLINE, SPORTDiscus, and OpenDissertations; Clinicaltrials.gov; Web of Science; Scopus; Cochrane Database of Systematic Reviews; and Physiotherapy Evidence Database (PEDro). The search encompassed articles written in English or Spanish and published between 1 January 2010 and 30 November 2022.

The search terms used were:

English: (physical activity or exercise or physical exercise or fitness or strength training or physical intervention or cointerventions) AND (pregnancy or pregnant or maternal or antenatal) AND (randomized clinical trial or RCT) AND (miscarriage).

Spanish: (actividad física OR ejercicio OR ejercicio físico OR fitness OR entrenamiento OR intervención física OR co-intervención AND embarazo OR embarazada OR maternal OR prenatal AND ensayo clínico aleatorizado AND aborto.

**Figure 1:**
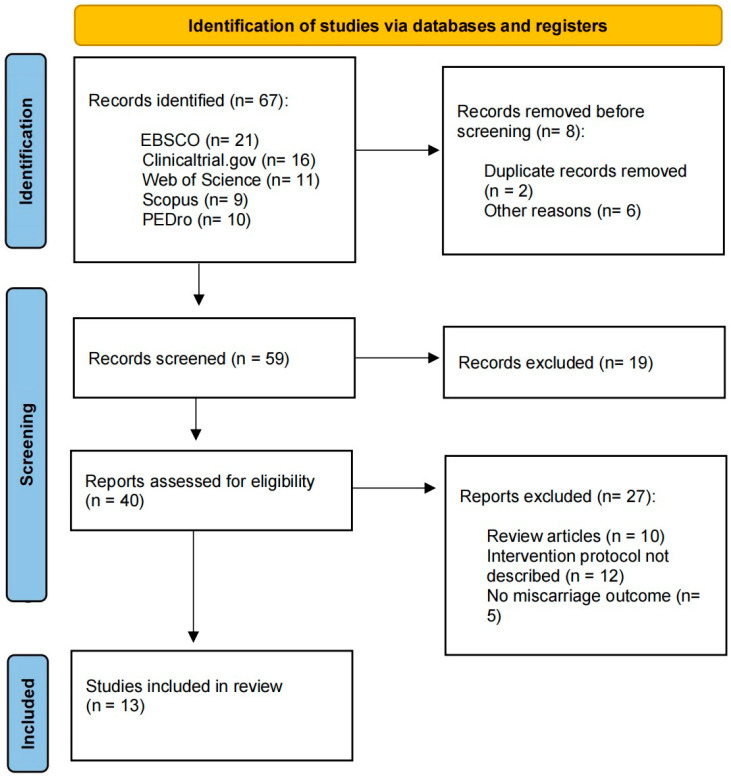
Flow diagram of the analyzed articles.

Regarding inclusion criteria, the eligible articles for review comprised studies that measured physical activity or exercise intervention (excluding articles that solely provided advice for an active pregnancy or those that included a measurable physical activity questionnaire but lacked an exercise intervention). The outcome of interest was miscarriage, and the characteristics of the physical activity or exercise program were also considered. This process is illustrated in Figure 1. Two reviewers (RB and CS) independently screened titles and abstracts of all identified citations and potentially eligible articles were selected. Full-text articles were independently assessed by the two reviewers for eligibility criteria. Any discrepancies were resolved by consensus.

Additionally, secondary outcomes such as physiological, sociodemographic, and delivery outcomes were examined by two reviewers (DZ and MS) to evaluate the effects of each intervention on maternal health. However, these secondary outcomes were not included in the meta-analyses. From each selected study, we extracted the following information: author(s), publication year, country where the study was conducted, study design type, number of participants, characteristics of the intervention program, and the variables analyzed (both primary and secondary outcomes).

### 2.6. Statistical Analysis, Quality of Evidence Assessment, and Risk of Bias

Meta-analyses were conducted with the dependent variable being the miscarriage ratio in each study, categorized as either miscarriage “yes” or “no”. The number of events observed in each study group and their respective relative risks (RR) were recorded. The total RR sum was calculated using a random effects model [11]. Each study was assigned a weight based on its sample size, contributing to the overall analysis and establishing a weighted average. The I^2^ statistic was utilized to quantify the heterogeneity observed in the results due to variations in interventions and study designs, indicating the extent of variability in the effects of each intervention, which were non-random.

The following criteria were employed to classify heterogeneity levels: low heterogeneity (25%), moderate heterogeneity (50%), and high heterogeneity (75%) [5,12]. In cases of high heterogeneity, one possible approach is to subgroup the studies based on different characteristics that may explain this variability. However, considering the limited results of our study, we deemed it more appropriate to include all articles in each analysis, thus providing a comprehensive.

Analyses were performed using Review Manager (RevMan computer program, 5.4 version).

The quality of evidence for the primary outcome and each individual study was assessed using the Grading of Recommendations Assessment, Development and Evaluation (GRADE) framework, with RCT studies rated as moderate or high quality [13]. To evaluate the potential risk of bias (including selection, performance, attrition, detection, and reporting bias), the Cochrane Handbook guidelines were followed [14]. Randomized clinical trials were initially considered to have a “low” risk of bias due to their study design and intervention, in comparison to non-randomized interventions. However, their risk of bias could be either increased or decreased depending on the presence of “high” or “low” scores across the different bias sources. Non-RCTs were rated lower due to study design and were rated as very low, low, or moderate [13].

**Table 1:** Characteristics of the studies included in the review.

Author	Year	Country	Type	N	EG	CG	Intervention, Physical Exercise Program							Main Variables Analyzed	Secondary Variables Analyzed
							Freq	Intensity	Duration of Program	Type of Exercise	Superv Class	Duration of Class	Adh.		
Brik [15]	2019	Spain	RCT	120	75	45	3	55–60% Max HR	29 w	Aerobic, strength, coordination and balance, and pelvic floor exercises	Yes	60 min	70%	Gestational weight gain, miscarriage	Type of delivery, birth weight, gestational age
Daly [16]	2017	Ireland	RCT	88	44	44	3	Mod	26 w	Aerobic, resistance, pelvic floor exercises	Yes	50–60 min	-	Maternal fasting plasma glucose, gestational weight gain	Type of delivery and, birth weight, miscarriage
Petrov Fieril [17]	2014	Sweden	RCT	92	51	41	2	Mod	12 w	Resistance training	Yes	60 min	-	Health-related quality of life, physical strength	Birth weight, gestational age, miscarriage
Garnaes [4]	2017	Norway	RCT	91	46	45	5	Mod	24 w	Endurance and strength training. Pelvic floor muscle exercises every day	Yes (3) No (2)	60 min 50 min	50%	Birth weight, gestational age miscarriage	Type of delivery
Guelfi [18]	2016	Australia	RCT	172	85	87	3	Mod	14 w	Home-based stationary cycling program	Yes	20–60 min	-	Gestational diabetes, miscarriage	Type of delivery, birth weight
Kluge [19]	2011	South Africa	RCT	50	26	24	7	Low–Mod	10 w	Five instruction exercise class + transverse abdominal and pelvic floor muscles training.	No	30 min	-	Pain intensity and functional ability	Type of delivery, birth weight, duration of labor, miscarriage
Navas [20]	2021	Spain	RCT	294	148	146	3	55–65% Max HR	20 w	Aquatic exercise	Yes	45 min	-	Postpartum depression, quality of life, and quality of sleep	Miscarriage, gestational age, and birth weight
Pelaez [6]	2019	Spain	RCT	345	115	230	3	65–70% Max HR	24 w	Aerobic and resistance training	Yes	60–65 min	80%	Gestational weight gain, macrosomia, type of delivery	Miscarriage
Renault [21]	2014	Denmark	RCT	283	142	141	7	Low	24 w	Daily 11,000 steps, hypocaloric low-fat diet	No	60 + min	-	Gestational weight gain, miscarriage	Gestational age
Sagedal [22]	2017	Norway	RCT	591	296	295	2	Mod	24 w	Aerobic, strength training. Dietary counselling	Yes	60 min	-	Gestational weight gain, birth weight	Gestational age, perineal tear, miscarriage
Ussher [23]	2015	UK	RCT	789	394	395	2–3	Mod	6 w	Treadmill exercise	Yes	20 + min	-	Continuous smoking abstinence, miscarriage	Type of delivery, birth weight
Vinter [5]	2011	Denmark	RCT	360	180	180	7	Mod	25 w	Aerobic and light strength training, + dietary counseling	Yes (1) No (6)	60 min 30–60 m	77%	Gestational weight gain, gestational diabetes	Type of delivery, birth weight, macrosomia, miscarriage
Wang [24]	2017	China	RCT	300	150	150	3	Mod	24 w	Stationary cycling program	Yes	45–60 min	-	Gestational diabetes, gestational weight gain	Birth weight, miscarriage

Author: first author last name, Year: year of study, Country: country where the article has been developed (usually in the method part), Type: randomized clinical (or controlled) trial. N: total number of women analyzed. EG: number of women analyzed in the intervention group. CG: Number of women analyzed in the control group. Freq: weekly frequency of exercise sessions. Intensity: moderate, high, low. Duration of program: weeks of duration. Type of exercise: aerobic, muscle strengthening, etc. Superv. Classes: whether or not there was supervision. Duration of class: minutes of each session. Adh.: adherence.

## 3. Results

### 3.1. Study Selection

A total of 67 articles were initially retrieved during the first stage of the search, and 8 were removed before screening: duplicate records removed (n = 2) and other reasons such as no pregnant population or lack of basic information (n = 6). Nineteen articles were then excluded because they did not meet the inclusion criteria. Subsequently, 27 articles were excluded for the following reasons: being a narrative review (n = 10), lacking a description of the intervention protocol (n = 12), or not providing information regarding miscarriage (n = 5). Ultimately, 13 RCT studies were included for further meta-analysis (Figure 1).

Regarding the type of intervention reported in the included studies (Table 1), the majority of them described physical activity sessions conducted by professionals in the respective field. These interventions encompassed various activities such as aerobic exercise, strength exercises, and aquatic activities, among others. The sessions outlined in the reviewed studies were designed to achieve moderate intensity and were conducted with a frequency ranging from one to seven days per week, lasting between 20 and 65 min per session. The duration of each intervention ranged from 2 to 24 weeks.

### 3.2. Effect of Physical Activity on the Occurrence of Miscarriage

**Figure 2:**
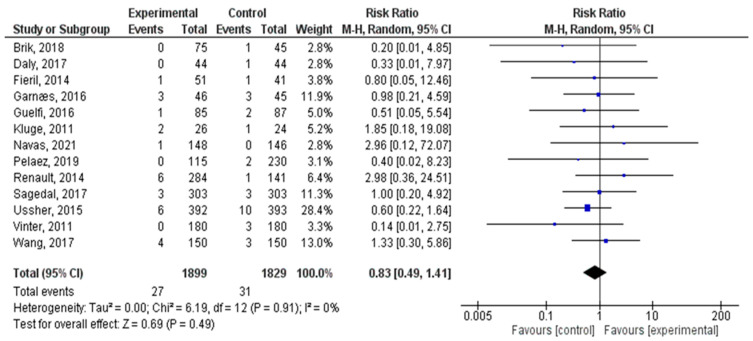
Effect of physical activity during pregnancy on miscarriage incidence [4,5,6,15,16,17,18,19,20,21,22,23,24].

A total of thirteen distinct RCTs were included in the present analysis, examining the incidence of miscarriage among women in both the experimental and control groups. The findings demonstrated no significant association between exercise practice during pregnancy and the occurrence of miscarriage (risk ratio = 0.83 95% CI = 0.83 (0.49–1.41); z = 0.69, *p* = 0.49; I^2^ = 0.00%, heterogeneity *p* = 0.91). Figure 2 illustrates the forest plot corresponding to the meta-analysis conducted.

### 3.3. Risk of Bias Assessment

**Figure 3:**
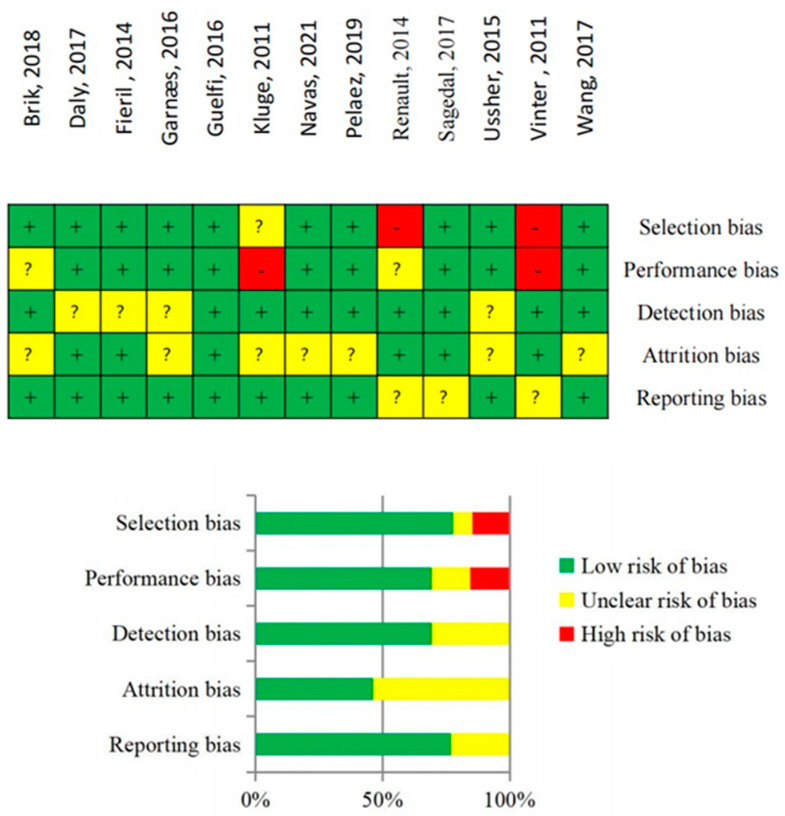
Risk of bias summary from the retrieved articles [4,5,6,15,16,17,18,19,20,21,22,23,24].

Overall, there was moderate quality evidence from 13 RCTs (n = 3728). The risk of bias in each article was rated as low, unclear, or high potential risk (Figure 3). The quality of evidence was downgraded due to selection bias [5,21] and performance bias [5,19]. Selection bias in this type of study mainly reflects the fact that randomization might have been compromised. Performance bias may be due to the fact that blinding participants is practically impossible in a controlled trial consisting of engaging in a physical activity program. More than half of the studies included in the review were rated as showing an unclear attrition bias, given the risk that participants who dropped out could have differed from those who remained in the study regarding factors associated with miscarriages. However, most of the studies were categorized as showing a low potential risk of bias.

## 4. Discussion

One of the barriers to being active during pregnancy is fear of harm to the baby and miscarriage [25,26]. The current study showed that exercise practice during pregnancy did not increase the risk of miscarriage with moderate quality evidence from 13 RCTs and 3728 women. We agree with the findings of a previous systematic review that examined the literature up to 6 January 2017 [3]. Our updated search including RCTs over the last 5 years confirms that pregnant individuals with no contraindications to exercise can exercise safely throughout pregnancy without fear of miscarriage, as there is no difference in miscarriage risk between those who exercise and those who do not.

Although we accepted all papers that listed miscarriage in the title or abstract, definitions of miscarriage varied between the 13 RCTs. A recent systematic review that examined specific first trimester risks of miscarriage (<14 weeks gestation) and exercise reported sparse and diverging results with only five studies and suggested that this was due to the wide heterogeneity of study design and exercise assessments between studies and the method of recall (either retrospectively after miscarriage or prospectively) [27].

Defining miscarriage precisely is a challenging task, primarily due to variations in the gestational period during which this complication can occur (measured in weeks of pregnancy) and the diverse and intricate approaches to the concept of miscarriage. The World Health Organization [28], as cited in a recent study on recurrent miscarriages [29], defines miscarriage as the expulsion or loss of a fetus at any point from conception to the 24th week of gestation. With this definition in mind, we found no risk to those who exercised prior to this time point.

The exercise intensity summarized in the current study ranges from low to moderate, with a mixture of exercise modalities, including walking, aquatics, aerobic activities, and resistance training, with 11 out of the 13 studies offering supervised exercise classes. The evidence would suggest that low- to moderate-intensity exercise does not increase the risk of miscarriage, however, studies regarding high-intensity exercise are limited. In individuals with in vitro fertilization who exercised more than 4 h per week, there was double the risk of miscarriage [30]. In addition, a large Danish study that assessed prenatal exercise in 90,000 women before and after having a miscarriage found a higher risk in those with a higher exercise volume of over 7 h per week [31]. However, when the authors only included those who were interviewed prospectively through a secondary analysis, the association was no longer significant [32]. More recently, when 34 international Norwegian athletes were examined and compared to active controls who engaged in greater than 150 min of exercise per week, there was no difference in miscarriage rates [33]. This was confirmed by Wowdzia et al. [34] in their systematic review and meta-analysis of elite athletes and pregnancy outcomes, where they concluded that there were no increased rates of miscarriage, although the quality of evidence was rated as “very low” due to inconsistency and risk of bias [34]. L’Heveder et al. [35] in their narrative review of pregnancy outcomes in elite sportswomen would concur that there is limited evidence; nevertheless, the existing data do not support an increase in adverse outcomes with increasing intensity. This would suggest that more studies are needed to evaluate the risk in those who exercise above the current pregnancy guidelines and that exercising within the guidelines in those without contraindications is considered safe. Furthermore, a recent systematic review that revisited absolute and relative contraindications to being physically active would suggest that recurrent miscarriage should be removed as a contraindication [36], as there is no scientific evidence that would suggest that exercising within the guidelines increases the risk of miscarriage, and by removing this contraindication, there may also be a removal of fear of miscarriage as a barrier to engaging in activity throughout pregnancy.

As exercise does not appear to be a risk factor for miscarriage, several authors have suggested that other biological, behavioral, and lifestyle factors may determine the risk of miscarriage [32,37]. These risk factors are multi-dimensional, complex, and not entirely understood, and include increased maternal age, infertility, alcohol consumption, smoking, and caffeine intake, although they remain controversial and unconfirmed [37]. Of note that may also increase the risk of miscarriage are previous pregnancy termination, stress, change in partner, and low pre-pregnancy weight [37]. Other factors include obesity, daily lifting of >20 kg, and working nights [32]. Evidence would suggest that advice encouraging a healthy diet, reducing stress, and promoting emotional well-being may help reduce miscarriage risk in early pregnancy [37].

The strengths of the current study are the use of methodological rigor (GRADE) to establish standards, our inclusion of randomized controlled trials, and the use of articles in English and Spanish. The limitations include the variability of miscarriage definitions between authors and the difficulty in standardizing exercise intensity and volume between studies.

## 5. Conclusions

“Moderate” quality of evidence showed no increase in miscarriage risk in those who engage in low- to moderate-intensity exercise compared to those who do not. More prospective research is needed to fully investigate the risks of miscarriage in those engaging in exercise above the current pregnancy guidelines.

## Data Availability

Not applicable.
